# Stabilization of the MAPK–Epigenetic Signaling Axis Underlies the Protective Effect of Thyme Oil Against Cadmium Stress in Root Meristem Cells of *Vicia faba*

**DOI:** 10.3390/ijms27010208

**Published:** 2025-12-24

**Authors:** Natalia Gocek-Szczurtek, Aneta Żabka, Mateusz Wróblewski, Justyna T. Polit

**Affiliations:** 1 Department of Cytophysiology, Faculty of Biology and Environmental Protection, University of Lodz, 90-236 Lodz, Poland; natalia.gocek.szczurtek@biol.uni.lodz.pl (N.G.-S.); aneta.zabka@biol.uni.lodz.pl (A.Ż.); mateusz.wroblewski@biol.uni.lodz.pl (M.W.); 2 Doctoral School of Exact and Natural Sciences, University of Lodz, 90-237 Lodz, Poland

**Keywords:** histone modification, DNA methylation, MAPK, transcription, cadmium, essential oil

## Abstract

Cadmium (Cd) induces oxidative stress and disrupts nuclear organization and chromatin-associated metabolic processes in plant cells. Therefore, identifying natural, biodegradable, non-bioaccumulative compounds that enhance plant tolerance to heavy metals is crucial. We hypothesized that Cd exposure (175 µM CdCl_2_, 24 h) activates mitogen-activated protein kinases (MAPKs), triggering defined epigenetic modifications that lead to transcriptional repression, and that thyme oil (TO; 0.03% (v/v), emulsified) mitigates these effects by stabilizing chromatin organization. We analyzed nuclear MAPK (p44/42) activation, global DNA methylation (5-methylcytosine; 5-mC), and selected histone modifications as key components of early stress signaling and epigenetic regulation. We found that Cd exposure doubled global 5-mC levels and caused pronounced alterations in histone marks, including decreases in H3K4Me2 (~34%), H3T45Ph (~48%), and H4K5Ac, accompanied by strong increases in H3K9Ac (~57%) and H3K56Ac (~148%). These changes were associated with chromatin condensation and reduced transcriptional activity. In contrast, co-treatment with TO maintained MAPK activity and epigenetic parameters close to control levels, preventing chromatin compaction and transcriptional repression. Together, these findings indicate that TO stabilizes the nuclear signaling–epigenetic interface under Cd stress and represents a promising bioprotective strategy. This work provides the first demonstration that TO modulates both MAPK activation and Cd-induced histone modifications in plants.

## 1. Introduction

Heavy metal pollution represents a major environmental and agricultural concern due to its toxicity to living organisms [1]. While some heavy metals, such as copper, zinc, and molybdenum, serve essential physiological roles as micronutrients [2], others—like cadmium, lead, and mercury—have no biological function and are toxic even at low concentrations, readily accumulating in plant and animal tissues [3]. Although naturally occurring in the Earth’s crust, the excessive environmental accumulation of heavy metals results primarily from anthropogenic activities, including industrial development, traffic, and the intensive use of fertilizers and agrochemicals. Among these metals, cadmium (Cd) is of particular concern due to its widespread occurrence and well-documented toxicity [4,5].

Cd is a highly mobile heavy metal in plants that readily accumulates in tissues and exerts pronounced phytotoxic effects. Its toxicity results from direct interference with key cellular structures and fundamental physiological processes, including disruption of water balance, ion transport, membrane integrity, protein synthesis and stability, as well as DNA integrity [6,7]. At the same time, Cd acts as a potent stress signal, inducing the production of multiple signaling molecules such as reactive oxygen species (ROS) [8,9,10], nitric oxide (NO), phytohormones, cyclic nucleotides, calcium ions and sugars. To cope with this complex spectrum of damage- and signal-derived cues, plant cells integrate environmental information with core metabolic pathways and activate coordinated protective responses aimed at maintaining cellular homeostasis and genome integrity [11,12,13,14,15,16,17,18,19].

A central element of this signaling network is the mitogen-activated protein kinase (MAPK) cascade, composed of sequentially acting MAPKKK → MAPKK → MAPK modules that enable efficient signal amplification [20]. Plant MAPKs are evolutionarily conserved enzymes, among which MPK3 and MPK6 (p44/42), homologues of animal extracellular signal-regulated kinases (ERK1/ERK2), are the most extensively characterized in stress responses [20,21]. Once activated, MAPKs phosphorylate a wide range of target proteins, modulating their activity, stability and subcellular localization [22]. When cytoplasmic responses are insufficient to ensure long-term adaptation, MAPK-mediated signaling extends to the nucleus, where phosphorylation of transcription factors links stress perception at the plasma membrane with transcriptional reprogramming. This results in the induction of defense-related genes [23,24,25] that can collectively enhance plant tolerance to heavy metal stress.

Increasing evidence indicates that MAPK pathways extend beyond transcription factor activation and are closely linked with chromatin-level regulation, including DNA methylation and post-translational histone modifications [26,27,28]. DNA methylation is generally associated with transcriptional silencing, particularly when occurring in promoter regions [29,30,31,32,33], while a dynamic balance between methylation and demethylation maintains epigenome stability and enables flexible gene regulation under stress [34,35]. In parallel, reversible histone modifications, including acetylation, methylation, and phosphorylation, alter DNA–histone interactions and modulate chromatin structure and gene function in a context-dependent manner, with their functional versatility arising from the diversity of modification sites and the number of attached groups [36,37,38,39,40,41,42,43,44,45,46,47]. The combinatorial interplay of histone marks, together with DNA methylation, forms a dynamic regulatory layer that fine-tunes transcription and enables plants to rapidly adjust gene expression in response to stress [48]. Although cadmium exposure induces significant epigenetic alterations, the mechanisms by which MAPK signaling links oxidative stress to chromatin remodeling in plants remain poorly understood.

In parallel with efforts to understand stress signaling mechanisms, increasing attention has been directed toward identifying natural compounds capable of enhancing plant tolerance to environmental stress [6,49]. Among such candidates, thyme oil (TO), widely used in traditional medicine, cosmetics, and the food industry, has attracted considerable interest due to its well-documented biological properties [50,51,52,53]. TO is rich in phenolic monoterpenes, primarily thymol and carvacrol, which exhibit strong antioxidant and anti-inflammatory activities [54,55]. In vitro studies have demonstrated that TO enhances the activity of key antioxidant enzymes, such as catalase and superoxide dismutase, thereby increasing overall cellular antioxidant capacity [56]. In addition, its broad-spectrum antimicrobial activity (antiviral, antibacterial, and antifungal) [57], together with the synergistic action of multiple components, makes TO an attractive natural protective agent. Previous studies indicates that TO can mitigate cadmium toxicity in animal models [54,58,59]; however, its potential as a bioprotective agent in plants exposed to heavy metals remains poorly explored, with only a limited number of studies addressing this aspect [60].

Building on these observations, our recent work demonstrated that TO, applied as a 0.03% (v/v) emulsion, significantly alleviates Cd-induced oxidative stress and genotoxic effect in root meristem cells of *V. faba* [55]. Given that cadmium exposure is known to disrupt chromatin organization and induce epigenetic alterations that may affect transcriptional activity, there is a strong rationale to investigate whether the protective effects of TO extend to the level of nuclear signaling and epigenetic regulation. To address this issue, we used primary roots of *Vicia faba* var. *minor*, a well-established model species in chromatin, cytogenetic, and epigenetic research. Its clearly defined meristematic zones, distinct nuclear morphology, and large chromosomes allow precise microscopic assessment of chromatin condensation, histone modifications, and nuclear signaling events. We incubated seedlings for 24 h in a solution of CdCl_2_ (175 μM), emulsified TO (0.03%), or a combination of both treatments. Compared with control seedlings incubated in water or in the emulsifier solution used to prepare the oil emulsion, we analyzed nuclear MAPK activity, followed by assessment of global DNA methylation as a classical marker of chromatin remodeling and evaluation of changes in the epigenetic patterns of selected histone marks (H3K4Me2, H3T45Ph, H4K5Ac, H3K9Ac, H3K56Ac), representing distinct regulatory mechanisms involved in the cellular stress response. Finally, we evaluated transcriptional activity.

Our study was designed to test two hypotheses: (1) Cd exposure activates nuclear MAPKs and induces specific epigenetic modifications that result in reduced transcriptional activity; and (2) TO mitigates Cd-induced effects by limiting epigenetic alterations and maintaining chromatin stability. Overall, this study provides the first evidence linking the protective effects of TO with modulation of nuclear MAPK signaling and epigenetic regulation in plants exposed to heavy metal stress.

## 2. Results

### 2.1. Immunodetection of Dually Phosphorylated MAPKs

To determine the localization and activity level of mitogen-activated protein kinases (MAPKs) in *V. faba* meristem cells, an immunocytochemical approach was employed using commercial anti-phospho-p44/42 monoclonal antibodies. In members of the Fabaceae family, these antibodies recognize several MAPK isoforms containing TEY (Thr-Glu-Tyr) and TDY (Thr-Asp-Tyr) motifs within their activation loops [61], most notably MPK3 and MPK6. These kinases are well-characterized stress-responsive MAPKs and functional homologs of mammalian ERK1/2 and act as central nodes within MAPK cascades, integrating signals from multiple stress-signaling pathways, which makes them particularly reliable markers of cascade activation [62,63].

**Figure 1:**
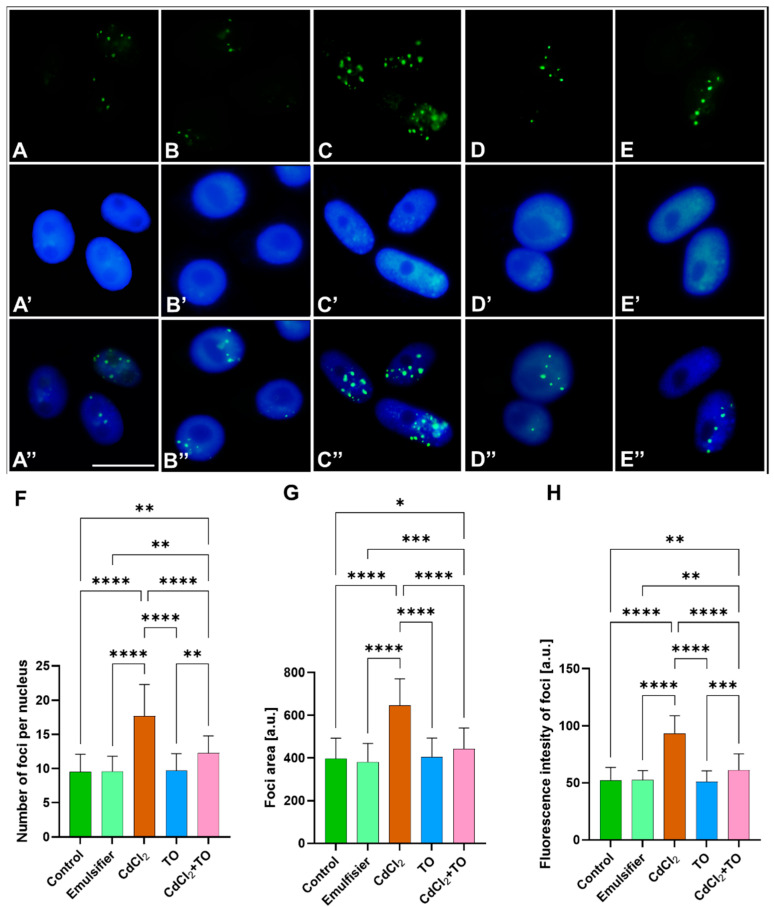
Immunofluorescence detection of dually phosphorylated MAPKs in primary root meristems cells of *V. faba* after 24 h of incubation with water—Control (**A**), emulsifier (**B**), CdCl_2_ (**C**), TO (**D**) and a combination of CdCl_2_ and TO (**E**). Corresponding images of cell nuclei stained with DAPI (**A’**–**E’**), and merged images of immunofluorescent signals with DAPI staining (**A”**–**E”**). Scale bar = 10 µm. Mean (± SD; *n* = 6 biological replicates) number of MAPK foci per nucleus; approximately 100 nuclei were analyzed per treatment (**F**). Mean (± SD; *n* = 6 biological replicates) area of MAPK foci; approximately 35 nuclei were analyzed per treatment (**G**). Mean (± SD; *n* = 6 biological replicates) fluorescence intensity of MAPK foci, expressed as pixel intensity (0–255); approximately 55 foci were analyzed per treatment (**H**). Statistical significance: **** *p* < 0.0001, *** *p* < 0.001, ** *p* < 0.01, * *p* < 0.05 (one-way ANOVA followed by Tukey’s multiple comparison test).

**Figure 2:**
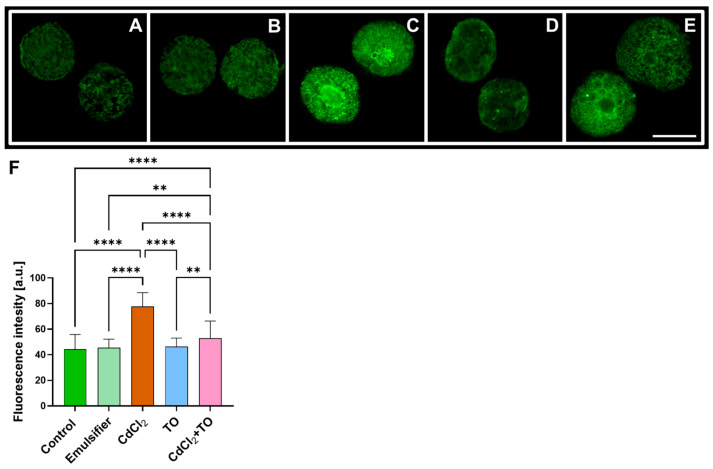
Immunofluorescence detection of 5-mC in nuclei of primary root meristem cells of *V. faba* after 24 h of incubation with water—Control (**A**), emulsifier (**B**), CdCl_2_ (**C**), TO (**D**), and a combination of CdCl_2_ and TO (**E**). Scale bar = 10 µm. Mean (± SD; *n* = 6 biological replicates) fluorescence intensity of 5-mC labeling; approximately 58 nuclei were analyzed per treatment (**F**). Statistical significance: **** *p* < 0.0001, ** *p* < 0.01 (one-way ANOVA followed by Tukey’s multiple comparison test).

**Figure 3:**
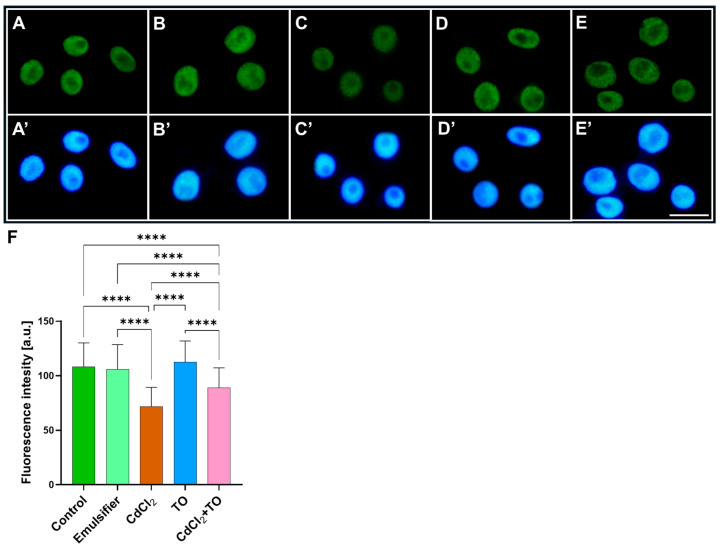
Immunofluorescence detection of H3K4Me2 in nuclei of primary root meristem cells of *V. faba* after 24 h incubation with water—Control (**A**), emulsifier (**B**), CdCl_2_ (**C**), TO (**D**), and a combination of CdCl_2_ and TO (**E**), along with corresponding images of cell nuclei stained with DAPI (**A’**–**E’**). Scale bar = 10 µm. Mean (± SD; *n* = 6 biological replicates) fluorescence intensity of H3K4Me2 labeling; approximately 120 nuclei were analyzed per treatment (**F**). Statistical significance: **** *p* < 0.0001 (one-way ANOVA followed by Tukey’s multiple comparison test).

Immunofluorescence signals were detected as distinct nuclear foci. Quantitative analysis was based on three parameters: (1) the number of foci per nucleus (Figure 1F), (2) the foci area (Figure 1G), and (3) fluorescence intensity (FI) (Figure 1H). The number of foci reflects the frequency of distinct, clearly separated chromatin-associated MAPK signaling microdomains. Foci area indicates the spatial extent of these domains, potentially corresponding to the size of chromatin region influenced by kinases. FI reflects the local accumulation of active MAPKs and relative activation levels within individual domains. Together, these parameters capture the complexity of nuclear MAPK signaling.

Control roots (Figure 1A), as well as those treated with the emulsifier (Figure 1B) or emulsified form of TO (Figure 1D), exhibited similarly low numbers of nuclear foci, averaging approximately 10 per nucleus (Figure 1F), which is consistent with basal MAPK activity. In contrast, CdCl_2_ treatment (Figure 1C) caused a significant, nearly twofold increase in foci number, indicating an expansion of functional MAPK microdomains. Co-treatment with CdCl_2_ and TO (Figure 1E) significantly reduced the number of foci relative to CdCl_2_ alone, suggesting partial normalization of nuclear MAPK domain frequency.

The mean foci area in control cells (Figure 1A), as well as in those exposed to the emulsifier (Figure 1B) and emulsified TO (Figure 1D), remained comparable (Figure 1G). CdCl_2_ exposure increased foci area by approximately 50% (Figure 1C), which is potentially consistent with an expansion of chromatin regions associated with active MAPKs or increased kinase density. In the combined CdCl_2_ and TO treatment (Figure 1E), foci area was only slightly higher than in controls, indicating partial preservation of domain size.

A similar pattern was observed for fluorescence intensity. Control, emulsifier-treated (Figure 1A,B) and TO-treated nuclei (Figure 1D) showed low FI values—around 50 a.u. per focus (Figure 1H). Exposure to CdCl_2_ (Figure 1C) induced a marked, nearly twofold increase in signal intensity, which may reflect enhanced local MAPK activity accumulation. In roots treated with combination of CdCl_2_ and TO (Figure 1E), FI increased only modestly relative to controls, suggesting intermediate MAPK activity per domain under these conditions.

### 2.2. Immunodetection of 5-Methylcytosine (5-mC)

Immunofluorescence labeling with specific antibodies enabled the visualization of DNA methylation levels (5-methylcytosine; 5-mC) across the nuclear chromatin of *V. faba* root meristem cells. Fluorescence intensity, which reflects the degree of DNA methylation and is generally associated with transcriptionally silent chromatin, varied depending on the experimental treatment (Figure 2A–F). In control roots, i.e., those incubated in water (Figure 2A) or in the emulsifier solution used for oil preparation (Figure 2B), fluorescence intensity remained similarly low, indicating relatively low levels of DNA methylation. In contrast, exposure to CdCl_2_ led to a pronounced, approximately twofold increase in signal intensity (Figure 2F). Treatment with TO alone produced fluorescence levels comparable to the control (Figure 2D). However, simultaneous exposure to TO and CdCl_2_ (Figure 2E) resulted in only a slight elevation in fluorescence intensity—and consequently in DNA methylation—relative to the control roots.

### 2.3. Dimethylation of Histone H3 on Lysine 4 (H3K4Me2)

After a 24 h incubation of primary *V. faba* roots in water (Figure 3A), an emulsifier solution (Figure 3B), CdCl_2_ (Figure 3C), TO (Figure 3D), or a combination of CdCl_2_ and TO (Figure 3E), root apical meristem cells were labeled with anti-H3K4Me2 antibodies, and fluorescence intensity (FI) across the entire nuclear area was quantified, as this modification produces a more diffuse and homogenous nuclear signal that does not form discrete foci suitable for count- or area-based analysis.

Compared with the control (Figure 3A) and emulsifier-treated roots (Figure 3B), exposure to CdCl_2_ (Figure 3C) resulted in an approximately 33% reduction in H3K4Me2 fluorescence intensity (Figure 3F), which may indicate a decrease in chromatin regions associated with this mark and a potential link to reduced transcriptional activity. A 24 h incubation of seedlings in TO solution alone did not cause significant changes relative to the control (Figure 3D,F), suggesting maintenance of baseline H3K4Me2-associated chromatin states. However, simultaneous treatment of plants with TO and CdCl_2_ led to a noticeable increase in fluorescence intensity compared with CdCl_2_ alone, although the signal remained below control levels (Figure 3E,F), which may reflect partial restoration of H3K4Me2 levels.

### 2.4. Phosphorylation of Histone H3 on Threonine 45 (H3T45Ph)

**Figure 4:**
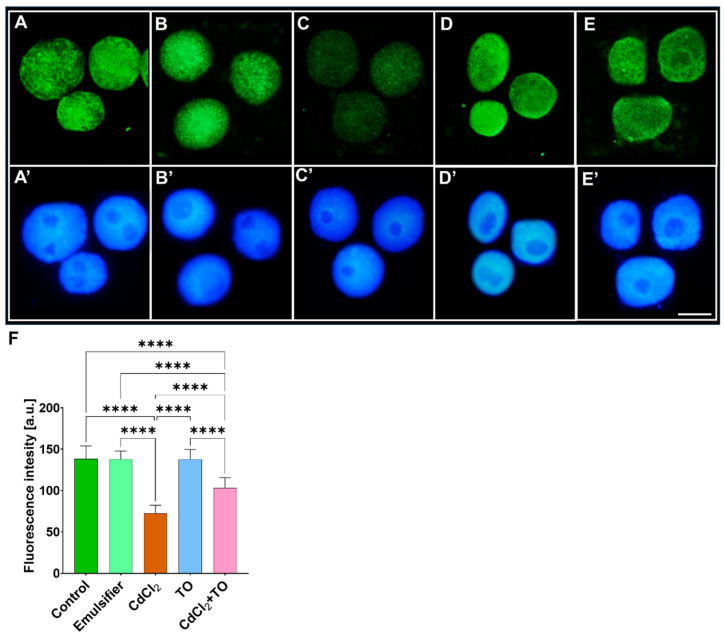
Immunofluorescence detection of H3T45Ph in nuclei of primary root meristem cells of *V. faba* after 24 h incubation with water—Control (**A**), emulsifier (**B**), CdCl_2_ (**C**), TO (**D**), and a combination of CdCl_2_ and TO (**E**), along with corresponding images of cell nuclei stained with DAPI (**A’**–**E’**). Scale bar = 10 µm. Mean (± SD; *n* = 6 biological replicates) fluorescence intensity of H3T45Ph labeling; approximately 50 nuclei were analyzed per treatment (**F**). Statistical significance: **** *p* < 0.0001 (one-way ANOVA followed by Tukey’s multiple comparison test).

Analysis of the relatively homogeneous fluorescence signal intensity obtained using specific antibodies against histone 3 phosphorylated at threonine 45 (H3T45Ph) revealed clear differences among the experimental series (Figure 4). Exposure to CdCl_2_ resulted in a marked decrease in H3T45Ph fluorescence intensity in interphase nuclei (Figure 4C), compared with roots incubated in water (Figure 4A), the emulsifier solution (Figure 4B), or the emulsified form of TO (Figure 4D). Such a reduction in H3T45Ph signal may indicate restricted nucleosome unwrapping dynamics, which can affect local DNA accessibility. After 24 h of simultaneous incubation of seedlings with CdCl_2_ and TO, a decrease in fluorescence intensity was also observed (Figure 4E), although the reduction was substantially smaller than in roots exposed to CdCl_2_ alone.

### 2.5. Acetylation of Histone H4 on Lysine 5 (H4K5Ac)

**Figure 5:**
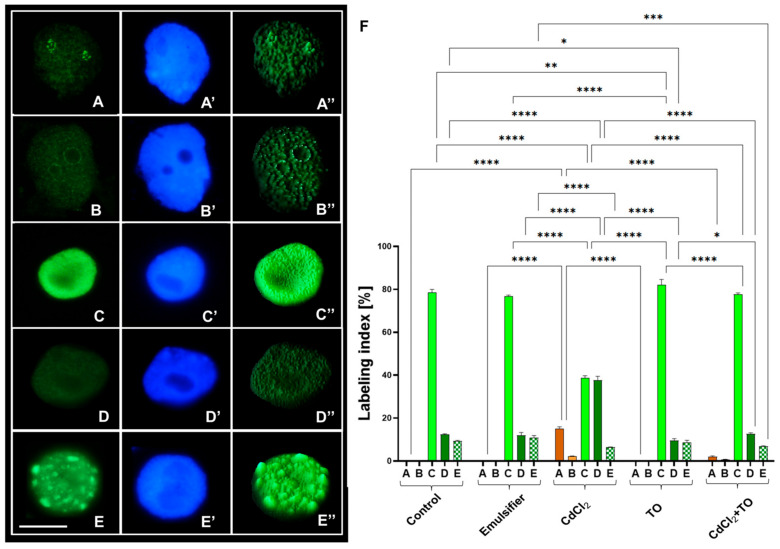
Representative immunofluorescence patterns of H4K5Ac labeling observed in nuclei of primary root meristem cells of *V. faba* after 24 h of treatment. Distinct labeling patterns were identified across all experimental variants and include: localization within the nucleolus (**A**), around nucleolar chromatin (**B**), strong euchromatic labeling (**C**), weak euchromatic labeling (**D**), and preferential heterochromatic labeling (**E**). Corresponding DAPI-stained nuclei (**A′**–**E′**), and interactive 3D surface plots (**A″**–**E″**) are shown. The frequencies (% ± SD; *n* = 3 biological replicates) of nuclei exhibiting each labeling pattern in control (water), emulsifier, CdCl_2_, TO, and a CdCl_2_ + TO treatments are quantified in panel (**F**). Scale bar = 10 µm. Statistical significance: **** *p* < 0.0001, *** *p* < 0.001, ** *p* < 0.01, * *p* < 0.05 (one-way ANOVA followed by Tukey’s multiple comparison test).

Immunodetection of histone H4 acetylation at lysine 5 (H4K5Ac) in interphase nuclei of *V. faba* root meristems revealed five distinct labeling patterns (Figure 5A–E), which were therefore analyzed separately. These included: 1. nuclei exhibiting homogeneous, strong labeling (Figure 5C); 2. nuclei with weaker labeling throughout the euchromatin region (Figure 5A,B,D); 3. nuclei displaying strong fluorescence within the nucleolus (Figure 5A); 4. nuclei showing fluorescence concentrated at the nucleolar periphery (Figure 5B); and 5. nuclei characterized by clustered fluorescence foci located in heterochromatin regions (Figure 5E). The frequency of these patterns varied among experimental treatment. Quantitative analysis therefore focused on determining the average percentage of interphase nuclei exhibiting each labeling type. It is worth noting that pronounced nuclear labeling is generally associated with increased chromatin accessibility, whereas weaker signals may reflect its partial silencing or condensation.

Labeling restricted to the nucleolus or its borders (Figure 5A,B) occurred exclusively in CdCl_2_ treated cells and, to a much lesser extent, in those incubated with the CdCl_2_ and TO mixture. This pattern was absent in control root meristem cells and in those incubated with emulsified TO (Figure 5F). Homogeneous, intense labeling across the chromatin (Figure 5C) represented the predominant pattern in roots treated for 24 h with water, the emulsifier solution, emulsified TO, and the CdCl_2_ + TO mixture. In contrast, seedlings exposed to CdCl_2_ alone showed a pronounced decrease in this labeling index (37.56%) relative to the control (73%). Conversely, the labeling type characterized by markedly weaker fluorescence (Figure 5D) accounted for approximately 35.5% of interphase nuclei in CdCl_2_-treated roots, whereas its frequency in the control, TO-treated, and CdCl_2_ + TO series did not exceed 13%. The final pattern, localized by heterochromatin region (Figure 5E), occurred at similar frequencies in control and TO-treated roots. Its incidence decreased following CdCl_2_ exposure. Supplementation with TO did not significantly modify the proportion of nuclei displaying this pattern relative to Cd-treated seedlings.

### 2.6. Acetylation of Histone H3 on Lysine 9 (H3K9Ac)

**Figure 6:**
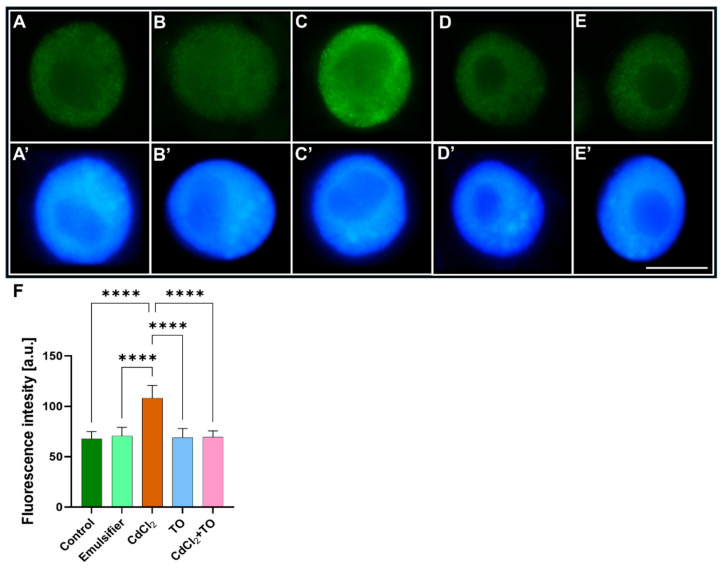
Immunofluorescence detection of H3K9Ac in nuclei of primary root meristem cells of *V. faba* after 24 h of incubation with water—Control (**A**), emulsifier (**B**), CdCl_2_ (**C**), TO (**D**), and a combination of CdCl_2_ and TO (**E**), along with corresponding images of cell nuclei stained with DAPI (**A’**–**E’**). Scale bar = 10 µm. Median (±CI; *n* = 6 biological replicates) intensity of H3K9Ac labeling; approximately 158 nuclei were analyzed in Control, 119 in emulsifier, 140 in CdCl_2_, 149 in TO, and 158 in CdCl_2_ + TO treatment groups (**F**). Statistical significance: **** *p* < 0.0001 (Kruskal–Wallis test with post hoc Dunn’s multiple comparison test).

Changes in histone H3 lysine 9 acetylation (H3K9Ac) under the experimental treatments were evaluated by measuring fluorescence intensity (FI) across entire nuclei, which was appropriate given the homogeneous labeling pattern obtained using immunocytochemical detection. Analyses of root meristem cells from the control groups, i.e., seedlings incubated in water (Figure 6A) or in the emulsifier solution (Figure 6B), revealed comparably low fluorescence levels. In contrast, exposure to CdCl_2_ (Figure 6C), increased fluorescence intensity by approximately one-third relative to the control (Figure 6F), which may reflect a chromatin configuration that enhances accessibility to promoters of stress-responsive genes. Incubation of roots in emulsified TO (Figure 6D) did not alter histone acetylation levels compared with the control. Likewise, seedlings simultaneously treated with CdCl_2_ and TO (Figure 6E) exhibited acetylation levels similar to those of the control.

### 2.7. Acetylation of Histone H3 on Lysine 56 (H3K56Ac)

**Figure 7:**
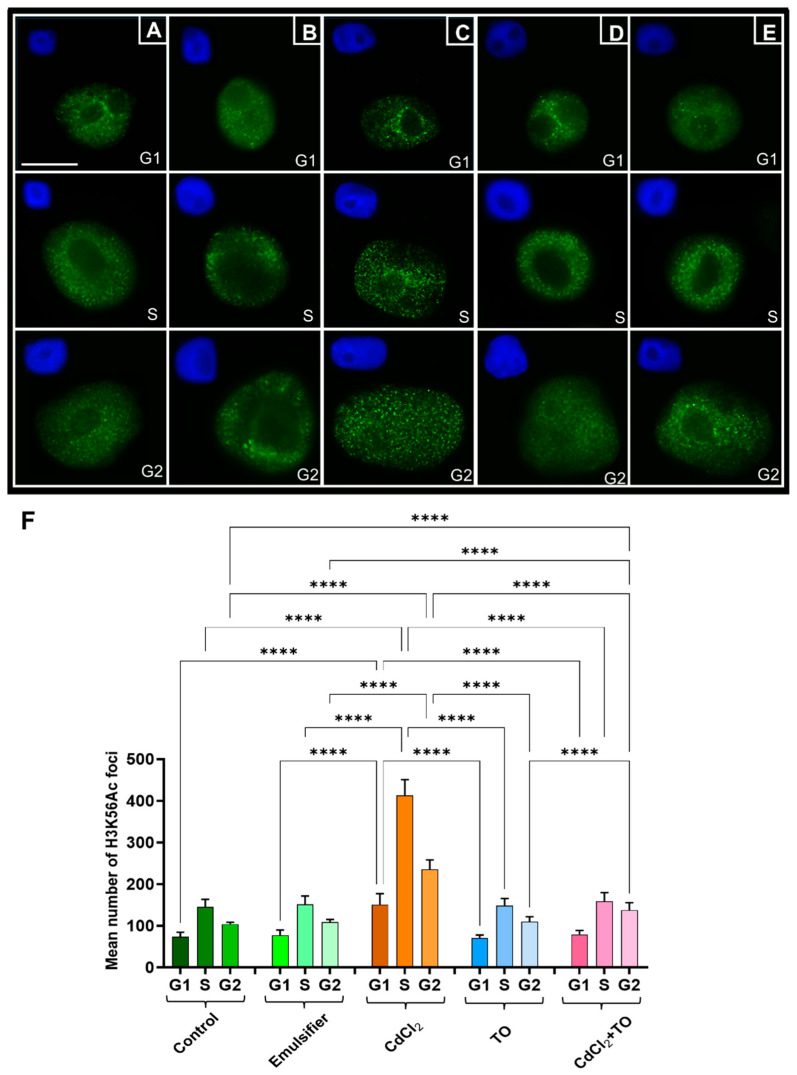
Immunofluorescence detection of H3K56Ac in G1-, S-, and G2-phase nuclei of primary root meristem cells of *V. faba* after 24 h incubation with water—Control (**A**), emulsifier (**B**), CdCl_2_ (**C**), TO (**D**), and a combination of CdCl_2_ and TO (**E**). Scale bar = 10 μm. Corresponding images of cell nuclei stained with DAPI, embedded in the upper left corners of each image. Quantitative analysis of the mean number of intracellular H3K56Ac foci in G1-, S-, and G2-phase nuclei for each treatment is presented in panel (**F**). Data are shown as mean (±SD; *n* = 6 biological replicates); approximately 90 nuclei were analyzed per treatment. Statistical significance: **** *p* < 0.0001 (one-way ANOVA followed by Tukey’s multiple comparison test).

To assess the effects of the examined treatments on histone H3 lysine 56 acetylation (H3K56Ac) in *V. faba* root meristem cells, immunocytochemical labeling was performed. Microscopic image analysis enabled quantitative evaluation of the average number of fluorescent foci in G_1_-, S-, and G_2_-phase nuclei under the applied experimental conditions (Figure 7A–E). Due to the small size and dense distribution of the fluorescent signals, their area and intensity were not analyzed, as such measurements were technically challenging and yielded unreliable results.

In the control groups, seedlings incubated in water (Figure 7A) or in the emulsifier solution (Figure 7B), the average number of H3K56Ac foci per nucleus was approximately 73, 145, and 104, and 78, 151, and 109 for G_1_, S, and G_2_ phases, respectively. Within each group, significant differences in foci number were observed between cell cycle phases, consistent with the dynamic incorporation of H3K56Ac into newly synthesized histones and its association with chromatin states permissive to DNA strand separation during replication. Exposure to CdCl_2_ (Figure 7C) led to a marked increase in H3K56 acetylation at all interphase stages. The number of foci approximately doubled in G_1_, nearly tripled in S, and increased more than twofold in G_2_ compared with controls (Figure 7F), reflecting enhanced chromatin relaxation potentially facilitating recruitment of DNA repair complexes. In TO-treated cells (Figure 7D), foci numbers in all phases remained comparable to controls. Simultaneous exposure to CdCl_2_ and TO (Figure 7E) significantly reduced H3K56 acetylation relative to CdCl_2_ alone. In G_1_ and S phases, foci numbers returned to control-like levels, whereas in G_2_, they remained lower than in CdCl_2_-treated seedlings but were still significantly higher than in the control group.

### 2.8. Changes in Transcription Dynamics

To evaluate transcriptional activity in the root meristem cells of *V. faba*, incorporation of the alkyne-modified uridine analog 5-ethynyluridine (EU) was used. This approach allows detection of newly synthesized RNA through a copper-catalyzed cycloaddition reaction, eliminating the need for immunocytochemical labeling. Quantitative analysis was performed by measuring average fluorescence intensity within nucleoplasmic and nucleolar regions.

**Figure 8:**
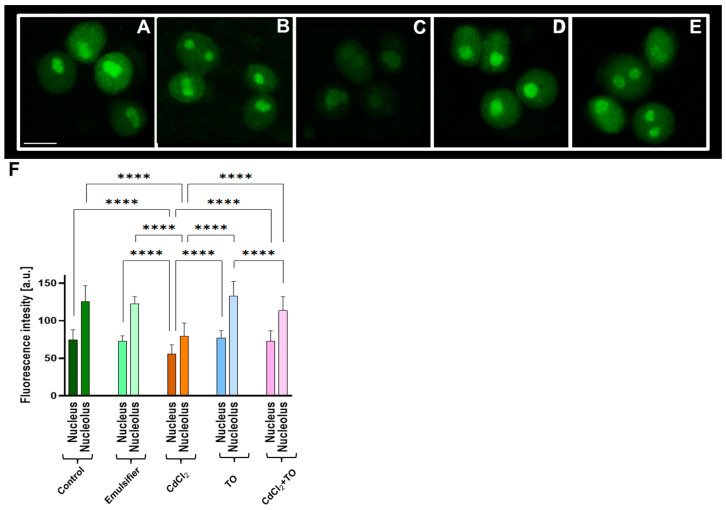
Detection of RNA synthesis using EU incorporation followed by Click-iT^®^ RNA Alexa Fluor^®^ 488 imaging in primary root meristem cells of *V. faba* after 24 h of incubation with water—Control (**A**), emulsifier (**B**), CdCl_2_ (**C**), TO (**D**), and a combination of CdCl_2_ and TO (**E**). Scale bar = 10 µm. Median (±CI; *n* = 6 biological replicates) EU labeling intensity (fluorescence intensity), expressed as a percentage of the maximum possible brightness (pixel value  =  255); approximately 250 nuclei were analyzed per treatment (**F**). Statistical significance: **** *p* < 0.0001 (Kruskal–Wallis test with post hoc Dunn’s multiple comparison test).

The strongest EU-derived fluorescence signals were detected in the nucleoli (Figure 8F), reflecting intense ribosomal RNA synthesis. Compared with cells incubated in water (Figure 8A) or emulsifier solution (Figure 8B), CdCl_2_ treatment caused a pronounced reduction in fluorescence intensity in both the nucleoplasm and nucleoli (Figure 8C,F), indicating decreased RNA synthesis under these conditions. Treatment with TO alone (Figure 8D,F) maintained transcriptional activity at levels comparable to controls. Notably, co-treatment with TO and CdCl_2_ (Figure 8E,F) substantially restored fluorescence intensity, reaching levels similar to controls and significantly higher than in CdCl_2_-only treated roots.

## 3. Discussion

Plants, due to their sessile lifestyle, have evolved sophisticated mechanisms for detecting environmental threats, including the presence of toxic heavy metals such as cadmium. These mechanisms are based on complex signaling networks that initiate defense and repair responses, as well as metabolic adjustments aimed at maintaining cellular homeostasis [62,63,64,65,66]. However, prolonged or high-intensity stress exposure can overwhelm these protective systems, leading to cellular dysfunctions that ultimately impair plant growth and development [67].

Consistent with such stress-induced cellular dysfunctions, exposure of *V. faba* seedlings to cadmium (175 µM CdCl_2_) resulted in a marked increase in genotoxicity, reflected by an increase in the proportion of cells exhibiting DNA damage (identified by γ-H2A.X labeling characteristic of DNA double-strand breaks) from approximately 1% to nearly 30% [55]. This effect was associated with enhanced oxidative stress, manifested by a fourfold increase in hydrogen peroxide (H_2_O_2_) levels and a twofold increase in superoxide anion (O_2_•^−^) content compared with control conditions [55].

Considering the need to identify natural and safe compounds capable of protecting plant cells against cadmium toxicity, particularly in the context of crop protection and yield stability, we further demonstrated that emulsified thyme oil (TO; 0.03%), rich in monoterpenes (thymol, *p*-cymene, carvacrol, limonene, and α-pinene), when applied simultaneously with cadmium, maintained ROS levels close to those of the control. As a result, TO effectively blocked the initiation of oxidative damage cascades, stabilized genome integrity, and reduced the proportion of cells exhibiting DNA double-strand breaks to approximately 5% [55].

Nevertheless, both heavy metals and ROS generated in their presence are potent activators of MAPK signaling cascades, which transmit stress-related information to the nucleus and initiate chromatin remodeling processes and stress-responsive gene regulation [26,27,28,68,69,70,71,72]. Since the mechanisms linking MAPK signaling with oxidative stress and chromatin regulation in plant cells remain insufficiently understood, in the present study we analyzed MAPK (p44/42) activation, global DNA methylation, histone modifications, and transcriptional activity. Together, all these analyses provide new insight into the relationship between MAPK signaling and epigenetic regulation under cadmium stress and demonstrate the ability of TO to modulate the plant cellular response pathway to cadmium stress.

### 3.1. Thyme Essential Oil Mitigates Cadmium-Induced Nuclear MAPK Activation

Cadmium exposure, along with increasing ROS levels in *V. faba* root meristem cells, leads to pronounced activation of nuclear MAPKs (MPK3/MPK6, homologs of ERK1/2 in animal cells). Immunocytochemical analyses revealed increases in the number, size, and intensity of MAPK-positive foci within chromatin, which is consistent with previous reports of cadmium-induced MAPK activation in *Medicago sativa* roots [13] and in *Oryza sativa* [73]. These results indicate that the applied Cd concentration activates a MAPK-dependent signaling pathway targeting the nucleus, thereby facilitating the initiation of defense mechanisms, including an epigenetic response. Such responses, mediated through chromatin reorganization, serve to protect DNA from endonuclease-mediated degradation [74] and enable adaptive transcriptional reprogramming [22,28].

A significant and, to our knowledge, previously unreported finding of this study is that TO prevents cadmium-induced activation of nuclear MAPKs in plant cells. While comparable plant studies are limited, similar effects of TO have been reported in animal models. Carvacrol, a major component of TO, inhibits p38 MAPK activity and reduces pro-inflammatory cytokine levels in the lungs of cadmium-exposed rats [75], and both thymol and carvacrol were shown to suppress c-Jun N-terminal kinase (JNK) activation [76]. Considering our findings that TO reduces ROS levels under cadmium stress [55], together with our unpublished data confirming a robust activation of antioxidant defenses by TO, it is reasonable to propose that the protective effect of TO arises primarily from its ability to attenuate oxidative stress, thereby limiting nuclear MAPK phosphorylation. Therefore, MAPK activity remains close to control levels, correlating with normal seedling growth despite cadmium exposure. Thus, TO effectively alleviates cadmium toxicity by restraining MAPK-dependent nuclear stress signaling. Nonetheless, a direct inhibitory effect of specific TO components on MAPKs, analogous to that observed in animal cells, cannot be excluded.

### 3.2. Thyme Essential Oil Attenuates Cadmium-Induced DNA Hypermethylation

DNA methylation is a crucial regulatory mechanism in plants, controlling development, cell differentiation, and stress responses [77,78,79], while also maintaining genome stability by silencing transposable elements [80]. Methylation of promoter regions typically leads to chromatin condensation and transcriptional repression by restricting transcription factor access [29,81]. In the present study, immunofluorescence-based analysis of 5-mC revealed a pronounced increase in DNA methylation across nuclear chromatin in *V. faba* root meristem cells following CdCl_2_ exposure. This hypermethylation coincided with enhanced nuclear MAPK activity, pointing to a functional link between stress-induced signaling and epigenetic regulation of the cellular response. Comparable cadmium-induced increases in global DNA methylation have been reported in soybean (*Glycine max*) and radish (*Raphanus sativus*), supporting the view that genome hypermethylation represents a conserved adaptive response to heavy metal stress in plants [82,83]. Importantly, co-treatment with Cd and TO effectively prevented the Cd-induced rise in 5-mC levels, which remained close to those observed in control cells. Given the established role of oxidative stress as an upstream driver of stress signaling and epigenetic remodeling, this effect is consistent with the ability of TO to attenuate ROS accumulation and limit the activation of downstream nuclear responses. In this context, the stabilization of DNA methylation patterns likely reflects a reduced propagation of stress signals toward chromatin remodeling pathways. This interpretation is further supported by our ongoing, unpublished analyses indicating enhanced antioxidant system activity in TO-treated seedlings under cadmium stress.

Although direct evidence for the impact of TO on DNA methylation in plants remains limited, studies in animal systems provide supportive parallels. Bozkurt et al. [84] demonstrated that *Thymus* spp. extracts reduce the expression of DNA methyltransferase 1 (DNMT1) in human cancer cells. Given that DNMT1 is functionally analogous to the plant maintenance methyltransferase 1 (MET1), responsible for preserving CG-context methylation [85], and that these enzymes share a high degree of homology within their catalytic domains [86], it is plausible that bioactive components of TO may also act at the enzymatic level in plant cells, modulating the DNA methylation machinery itself. Together, these observations suggest that TO-mediated modulation of DNA methylation represents an additional complementary layer of its protective action against cadmium-induced stress, beyond the upstream effects on ROS signaling pathway.

### 3.3. Thyme Essential Oil Modulates Cadmium-Induced Histone Modifications

Epigenetic DNA modifications act in concert with histone modifications to regulate chromatin accessibility for replication, repair, and transcription [87,88]. Abiotic stresses trigger dynamic changes in histone marks [89,90,91,92], allowing chromatin reorganization and gene expression reprogramming in response to DNA perturbations [93]. The direction and magnitude of these modifications, however, depend on species, tissue type, stressor, and metabolic context [91,94,95,96,97]. Our results indicate that cadmium strongly alters histone methylation, acetylation, and phosphorylation patterns in *V. faba* root meristem cells, underscoring the central role of epigenetic regulation in response to heavy metal stress.

A well-conserved mark is methylation of lysine 4 on histone H3 (H3K4), which can exist in mono-, di-, and trimethylated forms [98]. While H3K4Me1 is mostly within coding regions, H3K4Me2 and H3K4Me3 are enriched at promoters near transcription start sites, a pattern conserved across yeast [99], animals [100], and plants [101,102]. Although H3K4 methylation generally marks active chromatin, the functional role of H3K4Me2 remains debated [100,101,102,103]. In plants, H3K4Me2 is often associated with transcriptional repression [104,105,106], whereas in mammals, it protects active promoters from de novo DNA methylation by preventing DNMT3L binding, a cofactor of DNMT3A/B methyltransferases that preferentially interacts with unmethylated H3K4 [107,108]. Importantly, H3K4Me2 function is context-dependent, and our observations, based on correlative immunocytochemistry, reflect global patterns rather than gene-specific effects. The cadmium-induced decrease in H3K4Me2 in *V. faba* root meristem cells aligns with increased global DNA methylation and reduced transcriptional activity. This suggests that cadmium may compromise epigenetic regulation at active promoters in a way that is reminiscent of the protective function of H3K4Me2 in animal systems. Comparable decreases in H3K4Me2/3 have been reported in tea (*Camellia sinensis*) cells under drought stress [109], whereas increases in H3K4Me2 were reported in maize (*Zea mays*) following drought and salt stress [110]. Together, these observations provide insight into how cadmium disrupts histone-mediated chromatin regulation and set the stage for examining how TO may counteract such epigenetic perturbations.

Phosphorylation of histone H3 at threonine 45 (H3T45Ph), which participates in nucleosome unwrapping, can significantly influence local DNA accessibility to transcriptional, repair, and replication complexes [111]. The T45 residue of H3, together with neighboring Y41 and R42, is located near the DNA entry/exit site of the nucleosome and forms part of the so-called “H3 latch,” stabilizing DNA-histone interactions [112]. Phosphorylation of H3T45 changes the electrostatic charge of this residue, weakens its interaction with DNA, and facilitates the unwrapping of DNA ends, thereby promoting local chromatin relaxation. Although this modification remains poorly characterized in plants, studies in budding yeast (*Saccharomyces cerevisiae*) [113,114] and in HeLa cells [115] demonstrate its involvement in chromatin remodeling during DNA replication and the DNA damage response. Our results show that H3T45Ph levels were significantly reduced in *V. faba* meristem cells following cadmium exposure, consistent with the observed increase in DNA methylation and the overall reduction in transcriptional activity.

A similar conclusion arises from the analysis of histone H4 acetylation at lysine 5 (H4K5Ac). Under physiological conditions, H4K5Ac is abundant in interphase cells, present in both euchromatic and heterochromatic domains, and generally associated with chromatin relaxation required for transcription and replication [90]. Our immunocytochemical analyses revealed a significant decrease in H4K5Ac labeling intensity following cadmium exposure. This reduction correlates with diminished replication activity, the presence of DNA damage indicated by γH2AX immunostaining [55], and the overall reduction in transcriptional activity observed in this study. Together, these results support the conclusion that cadmium triggers a broad chromatin-silencing response. Similar decreases in H4K5Ac under abiotic stress have been reported in kenaf (*Hibiscus cannabinus*) subjected to NaCl and polyethylene glycol (PEG) treatments [116].

H4K5 acetylation is also linked to gene “priming,” a preparatory marking of loci for rapid transcriptional activation in response to environmental cues [117]. This mechanism, observed in plant responses to abiotic stresses, may explain the strong H4K5Ac signal detected in a subset of nucleoli-associated nuclei, despite the overall reduction in this modification in cadmium-treated cells. The nucleolus, responsible for rDNA transcription and ribosome biogenesis, is particularly sensitive to stress and responds through chromatin reorganization and transcriptional changes [118,119]. In this context, local enrichment of H4K5Ac within nucleolar regions may reflect an adaptive mechanism to sustain rDNA transcription and ribosome production under cadmium-induced oxidative stress and disrupted cellular homeostasis. The nucleolar labeling pattern may also indicate selective activation of genomic regions critical for the stress response. Considering the concurrent increase in global DNA methylation and the decrease in the potentially activating histone mark H3K4Me2, the presence of local H4K5Ac signals likely represents an effort to maintain expression of genes essential for cell survival. This interpretation is supported by Yue et al. [120], who showed that heat-stressed *Zea mays* cells exhibit nucleolar reorganization accompanied by increased histone acetylation within the 45S rDNA region, correlating with elevated pre-rRNA synthesis. Similarly, in pak choi (*Brassica rapa* subsp. *chinensis*) exposed to cadmium, enhanced H4K5Ac levels within the 45S rDNA promoter were associated with activation of rRNA transcription [121].

Although CdCl_2_-induced stress generally decreases histone modifications associated with chromatin relaxation (H3K4Me2, H3T45Ph, H4K5Ac), we observed a concurrent increase in specific marks that accompany transcriptional activity under environmental stress. One such modification is the well-documented acetylation of histone H3 at lysine 9 (H3K9Ac), which in plants is enriched within promoter regions of genes activated by abiotic stressors [122]. In the context of heavy metal-induced oxidative stress, H3K9Ac may contribute to transcriptional reprogramming, enabling the rapid and selective induction of defense-related genes [123], including those encoding antioxidant proteins or detoxification enzymes (e.g., phytochelatin synthases), despite the overall transcriptional repression observed at the whole-nucleus level. This interpretation aligns with the findings of Wang et al. [124], who reported increased H3K9Ac accompanied by reduced histone methylation in plants exposed to heat stress, suggesting an epigenetic mechanism that supports dynamic transcriptional shift under changing environmental conditions. Similar observations were made in *Arabidopsis thaliana*, where elevated H3K9Ac levels in the promoters of stress-inducible genes such as *AtMYB29* and *AtGST1* correlated with their enhanced transcription [125]. Salt stress likewise promotes H3K9 acetylation: in *Oryza sativa*, severe salinity induced a pronounced increase in H3K9Ac, particularly in seedling leaves, with enrichment localized to promoters of salt-responsive genes [126]. Together with the previously discussed decreases in H3K4Me2, H3T45Ph, and H4K5Ac, these observations highlight a complex, locus-specific epigenetic response, in which certain stress-responsive genes remain transcriptionally active despite global chromatin condensation triggered by cadmium exposure.

The second histone modification that markedly increased following CdCl_2_ treatment was the acetylation of lysine 56 on histone H3 (H3K56Ac), a well-established marker of the cellular response to DNA damage. While this mechanism is well characterized in yeast [127], its role in plants remains less understood [128]. H3K56Ac facilitates chromatin relaxation, supports DNA strand separation, and promotes recruitment of DNA repair complexes [127]. It is also associated with newly synthesized histone H3 incorporated during DNA replication [129]. In this study, cadmium exposure strongly induced H3K56Ac, particularly in S-phase cells. This increase should be considered part of the broader cellular response to compromised genomic integrity, consistent with previous reports of cadmium’s genotoxic effects [55,90]. The functional importance of this modification for cell survival under stress is further supported by studies in *S. cerevisiae*, where mutation of lysine 56, preventing its acetylation, drastically reduces cell viability and renders cells hypersensitive to DNA damage and replication stress [130]. Collectively, these findings emphasize the essential role of H3K56 acetylation in maintaining genome stability and supporting adaptive stress responses, including in plant systems.

Many of the observed epigenetic changes—such as increased DNA methylation and reduced levels of H3K4Me2, H3T45Ph, and H4K5Ac—appear to represent elements of a coordinated response to cadmium stress, consistent with oxidative stress-driven signaling pathways, including ROS-dependent MAPK activation [69,131,132]. At the same time, these changes cannot be interpreted exclusively as regulated adaptive responses. Cadmium is a well-known inhibitor of thiol-containing proteins [133,134], and numerous epigenetic enzymes, including histone acetyltransferases, methyltransferases, and kinases, contain cysteine residues within their catalytic domains [135,136]. Therefore, the cadmium-induced reduction in activating histone marks, accompanied by transcriptional repression, may reflect not only stress signaling-mediated chromatin remodeling but also direct toxic interference with enzymatic activity, resulting in a partial “freezing” of epigenetic states. Under prolonged heavy metal stress, both adaptive and damage-related mechanisms are likely to coexist and interact.

**Figure 9:**
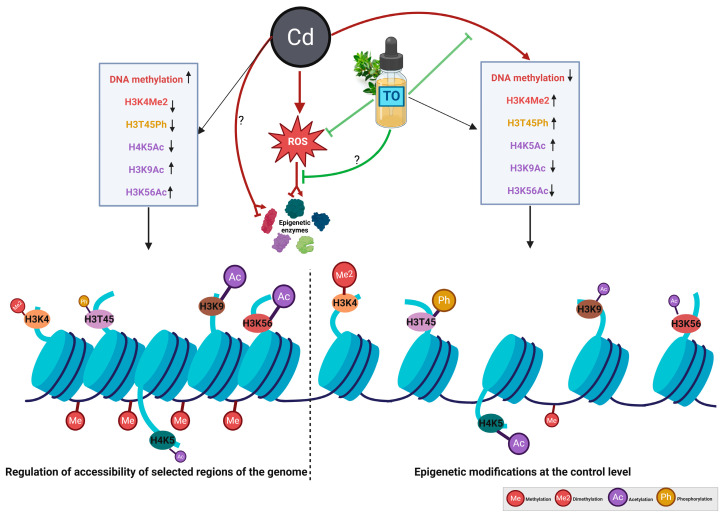
Schematic representation of the proposed mechanism illustrating the potential effects of cadmium (Cd) and thyme oil (TO) on epigenetic modifications. Arrows with sharp tips indicate activation or increase in enzymatic activity; arrows with flat tips indicate inhibition or reduction; arrows with both tips indicate that a factor can simultaneously activate some enzymes and inhibit others depending on the specific epigenetic mark. Cd exposure may induce oxidative stress (ROS), potentially affecting epigenetic enzyme activity and leading to changes in DNA methylation and histone modifications (↓H3K4Me2, ↓H3T45Ph, ↓H4K5Ac, ↑H3K9Ac, ↑H3K56Ac; arrows indicate increase [↑] or decrease [↓]). TO may counteract these effects by reducing oxidative stress, thereby restoring the histone modification profile (↓DNA methylation, ↑H3K4Me2, ↑H3T45Ph, ↑H4K5Ac, ↓H3K9Ac, ↓H3K56Ac). The bottom panel illustrates chromatin structural changes, highlighting potential regulation of genome accessibility under Cd alone or Cd combined with TO. This model is proposed and requires further functional studies for full confirmation. Created with BioRender.com.

Importantly, the heterogeneous nature of the observed modifications indicates that cadmium does not simple induce uniform chromatin silencing. Instead, it appears to promote selective reorganization of chromatin accessibility, in which general transcriptional programs are suppressed while stress-responsive loci may remain permissive or even activated. Although this selective regulation cannot be conclusively demonstrated without locus-specific analyses, the observed pattern is consistent with a stress-adaptive redistribution of transcriptional activity. Against this background, a key finding of the present study is that concurrent application of thyme essential oil effectively counteracts cadmium-induced epigenetic alterations. TO consistently modulated histone modifications in the opposite direction to cadmium exposure: it restored marks associated with chromatin relaxation (H3K4Me2, H3T45Ph, and H4K5Ac) toward control levels and limited the excessive accumulation of stress-associated modifications (H3K9Ac, H3K56Ac). Although TO did not always fully normalize all parameters, the overall epigenetic profile clearly antagonized the effects of CdCl_2_. To our knowledge, this study provides the first evidence that a plant essential oil can mitigate heavy metal toxicity by simultaneously modulating nuclear MAPK activity and multiple histone modifications, thereby stabilizing chromatin architecture and supporting transcriptional balance. The protective action of TO at the epigenomic level should be considered in a broader mechanistic context. Previous work demonstrated that TO reduces cadmium-induced ROS overproduction [55] and prevents excessive activation of nuclear MAPKs. Together with the present findings, this supports a model in which TO acts primarily at early stages of the stress response by limiting oxidative stress, which represents a major upstream trigger of MAPK signaling and downstream epigenetic remodeling (Figure 9). Similar protective mechanism have been described for *Rosmarinus officinalis* essential oil, which enhanced salt stress tolerance in durum wheat (*Triticum durum*) through ROS detoxification and stimulation of antioxidant defenses [137]. Collectively, these observations support the emerging view that plant essential oils can function as natural modulators of stress signaling and chromatin dynamics.

Finally, it cannot be excluded that cadmium-induced epigenetic responses in root meristem cells are also influenced by additional stress-related signals not directly linked to oxidative stress. Cadmium may impair the mobilization of endogenous reserves during early seedling development, including nitrogen pools, potentially activating alternative signaling pathways [138,139]. MAPK cascades are known to integrate diverse abiotic and metabolic cues [62,64], including nitrogen availability. For instance, RAF-like kinases at the MAPKKK level have been shown to respond to nitrogen status in green algae, illustrating how MAPK pathways can coordinate stress signaling with metabolic regulation [140]. Moreover, nitrogen deficiency has been reported to influence both MAPK activity and epigenetic modification dynamics in plants [141]. While these interactions remain speculative in the context of the present study, they point to additional regulatory layers that may shape chromatin responses under complex stress conditions and warrant targeted investigation in future work.

### 3.4. Thyme Essential Oil Preserves Transcription Under Cadmium Stress

The epigenetic alterations described above provide a mechanistic framework for the transcriptional changes observed under cadmium stress, through their impact on chromatin accessibility [142,143,144].

Using EU incorporation and fluorescence microscopy, we observed a pronounced reduction in RNA synthesis in cadmium-treated cells, affecting both the nucleoplasm and the nucleolus. This finding is consistent with previous reports demonstrating that cadmium exposure leads to widespread transcriptional disturbances, typically characterized by global repression of gene expression. For example, Kovalchuk et al. [145] showed that chronic exposure of plants to 50 μM Cd altered the expression of 403 genes, with 65 upregulated and 338 downregulated. Similarly, in barley (*Hordeum vulgare*), cadmium treatment (80 μM) markedly reduced transcript levels in both roots and shoots [146]. Such transcriptional suppression is a common feature of stress responses, in which energy-demanding growth-related programs are attenuated while resources are redirected toward defense and survival. At the same time, transcriptional regulation under stress is not uniform. Epigenetic modifications that repress and activate transcription can coexist within the same nucleus, reflecting the selective nature of stress responses [147]. While DNA methylation and loss of activating histone marks may silence genes involved in growth and development, stress-responsive loci can remain transcriptionally active or even be induced, for instance through histone acetylation such as H3K9Ac [148]. This model is supported by transcriptomic meta-analyses in rice, which revealed repression of photosynthesis-related pathways alongside activation of phytohormone- and stress-related genes under diverse stress conditions [149].

Importantly, our results show that TO effectively counteracts cadmium-induced transcriptional repression, restoring RNA synthesis to levels close to those observed in control cells. To our knowledge, this study provides the first evidence that plant essential oil can preserve transcriptional activity under heavy metal stress by simultaneously modulating MAPK signaling and epigenetic states thereby stabilizing chromatin organization. Although direct studies on the effect of TO on global transcription in plants are lacking, supportive data from animal systems indicate its broader regulatory potential. For instance, Firmino et al. [150] demonstrated that dietary supplementation with carvacrol- and thymol-containing blends significantly altered gene expression profiles in gilthead seabream (*Sparus aurata*), affecting 534 transcripts (393 upregulated and 141 downregulated). In *V. faba* the protective effect of TO on transcription likely represents a downstream consequence of its action at earlier stages of the stress response. By limiting oxidative stress and excessive MAPK activation, TO attenuates cadmium-induced epigenetic perturbations, preserves nucleolar integrity, and ultimately maintains transcriptional activity near physiological levels. Together, these findings place transcriptional preservation as the functional outcome of TO-mediated stabilization of the MAPK–epigenetic–chromatin axis under cadmium stress.

## 4. Materials and Methods

### 4.1. Plant Material

Sterile seeds of cultivated field bean (*Vicia faba* var. *minor*) (W. Legutko The Seed Breeding Company in Jutrosin, Poland) were sown in trays on moist blotting paper and germinated in darkness at 20 °C. After 72 h, seedlings with primary roots approximately 2–3 cm long were selected for uniform size, divided into experimental series and incubated for 24 h in the following media: 1—distilled water (control), 2—emulsifier solution used to prepare the essential oil emulsion, 3—cadmium chloride (CdCl_2_) solution at a concentration of 175 µM, 4—emulsified thyme oil (TO; Etja, Elbląg, Poland; composition previously analyzed by GC-MS [55]) solution at a concentration of 0.03% and 5—CdCl_2_ solution supplemented with emulsified TO. The CdCl_2_ concentration was selected based on available literature data and preliminary experiments testing a concentration range of 100–200 µM [55,90,151,152,153]. The concentration of commercially accessible TO was determined based on preliminary tests within the 0.01–0.06% range and previously published studies [55]. For proper dispersion of TO in water, it was emulsified using a mixture of mono- and di-ethoxylated C14-18 and unsaturated C16-18 glycerides, together with ethoxylated *Brassica napus* oil. The emulsification process involved mixing the emulsifier with TO at a 1:4 (v/v) ratio (resulting in a final emulsifier concentration of 0.0075% v/v), followed by vigorous shaking with a vortex mixer for 15 min. The emulsifier was provided by the Department of Agronomy, University of Life Sciences in Poznań. The experiments were conducted on three independently prepared cultures. In each of them, five experimental series were prepared on separate Petri dishes (3 cm high, 25 cm in diameter), each containing 10 seedlings. Two seedlings were collected from each dish for each analysis. As a result, *n* = 6 biological replicates (root meristems) were obtained for each condition, except for the H4K5Ac analysis which was performed on 3 biological replicates (3 root meristems).

### 4.2. Immunocytochemical Detection of Dually Phosphorylated MAPKs

For immunocytochemical detection of dually phosphorylated mitogen-activated protein kinases (MAPKs), excised 1.5 mm long *V. faba* root tips were fixed for 45 min at 4 °C in 4% paraformaldehyde buffered with PBS, washed with PBS and then incubated for 45 min in citrate-buffered 2.5% pectinase (pH 5.0; 40 °C). After rinsing with PBS, root meristems were crushed on Super Frost Plus slides (Menzel-Gläser Thermo Fisher Scientific, Waltham, MA, USA) in a drop of distilled water. Following freezing on dry ice, coverslips were removed, the slides were washed with distilled water, and air dried. The preparations were then treated for 50 min with blocking buffer (8% BSA (Sigma-Aldrich, St. Louis, MO, USA) and 0.1% Triton X-100 (Sigma-Aldrich), PBS; 20 °C), followed by incubation with primary monoclonal anti-phospho-p44/42 MAPK antibodies, which cross-reacts with plant homologs MPK3 and MPK6 [61] (1:200, Cell Signaling Technology, Danvers, MA, USA) diluted in the antibody dilution buffer (1% w/v BSA, 0.3% v/v Triton X-100, PBS). After overnight incubation (4 °C), the slides were washed with PBS and incubated for 90 min with secondary Alexa Fluor 488-conjugated anti-rabbit IgG antibodies (1:1000, Sigma-Aldrich; 20 °C) diluted in the same antibody buffer. DNA was counterstained for 15 min with 5 µM DAPI (Sigma-Aldrich), followed by washing in PBS. Finally slides were mounted in a PBS/glycerol mixture (9:1) containing 2.5% 1,4-diazabicyclo [2.2.2]octane (DABCO).

### 4.3. Immunocytochemical Staining for DNA Methylation

Apical parts of *V. faba* primary roots were fixed for 10 min in freshly prepared 4% paraformaldehyde buffered with PBS (4 °C). After fixation, the root tips were washed with cold Tris buffer (10 mM Tris (hydroxy-methyl)-aminomethane, 10 mM EDTA-2Na, 100 mM NaCI; pH 7.2). Cell nuclei were then isolated, dropped onto Super Frost Plus slides (Menzel-Gläser) and air-dried (according to Żabka et al. [153]. Slides were treated with 4 M HCl for 90 min (20 °C) to partially denature the nuclear DNA, washed with Tris buffer containing 0.5% Triton X-100 (pH 7.4), and incubated with primary monoclonal anti-5-methylcytosine (5-mC, Sigma-Aldrich) antibodies dissolved in the antibody dilution buffer (1% w/v BSA, 0.3% v/v Triton X-100, PBS). Following an 16 h incubation (4 °C), slides were washed with Tris buffer and incubated for 90 min with the secondary goat anti-rabbit Alexa Fluor^®^488-conjugated antibodies (1:500; Cell Signaling, Warsaw, Poland) dissolved in the antibody dilution buffer (1% w/v BSA, 0.3% v/v Triton X-100, PBS; 20 °C), washed in Tris buffer, and embedded in PBS mixture/glycerol (9:1) with 2.3% DABCO.

### 4.4. Immunocytochemical Detection of Histone Methylation, Acetylation and Phosphorylation

Excised root tips of *V. faba* were fixed for 10 min in freshly prepared PBS-buffered 4% paraformaldehyde (4 °C) and washed with cold PBS. The excised root tips were then squeezed in a drop of cold PBS buffer between two microscope slides to collect nuclear samples, which were dropped onto Super Frost Plus slides (Menzel-Gläser) and air-dried. The slides were then pre-treated for 90 min with blocking buffer (8% BSA and 0.1% Triton X-100, PBS; 20 °C), rinsed 3 times with PBS buffer and incubated overnight in a humid atmosphere (4 °C) with primary antibodies against one of the following histone modifications: H3K4Me2 (rabbit polyclonal, 1:200), H3K56Ac (rabbit monoclonal, 1:200), H3T45Ph (rabbit polyclonal, 1:100), H3K9Ac (rabbit monoclonal, 1:200), or H4K5Ac (rabbit monoclonal, 1:200) (all from Cell Signaling). After washing with PBS, the slides were incubated for 90 min with the secondary Alexa Fluor 488-conjugated anti-rabbit IgG (1:1000, Sigma-Aldrich; 20 °C) and counterstained for 15 min with 5 µM DAPI (Sigma-Aldrich; 20 °C). Slides washed with PBS were air dried and embedded in PBS mixture/glycerol (9:1) with 2.3% DABCO.

### 4.5. 5-Ethynyl Uridine (EU) Labeling and Visualization of Nascent RNA

*V. faba* seedlings were pulsed for 60 min with a 1 mM solution of 5-ethynyl uridine (5-EU; Thermo Fisher Scientific, Waltham, MA, USA) in the dark (20 °C). Then, excised meristems of 1.5 mm length were fixed for 45 min in PBS-buffered 4% paraformaldehyde (4 °C; pH 7.4), washed with PBS and incubated for 45 min with the citrate-buffered 2.5% pectinase (pH 5.0; 40 °C). After maceration, root meristems were washed with cold PBS, squashed onto Super Frost Plus slides (Menzel-Gläser), frozen with dry ice, washed with PBS and air dried. The slides were then permeabilized for 15 min with PBS containing 0.5% Triton X-100. Nascent RNA was visualized using Click-iT^®^ RNA Alexa Fluor^®^ 488 Imaging Kit with the reaction cocktail (Thermo Fisher Scientific). Following 60 min incubation (20 °C), slides were washed in Click-iT^®^ reaction rinse buffer and PBS. DNA was stained for 15 min with 5 µM DAPI (Sigma-Aldrich), and then the slides were washed in PBS. Specimens were mounted in PBS mixture/glycerol (9:1) with 2.3% DABCO.

### 4.6. Microscopic Measurements, Observations, and Analyses

Observations were performed with a Nikon Eclipse E600W fluorescence microscope (Nikon, Tokyo, Japan) featuring phase-contrast optics, a U2 filter (UVB light; λ = 340–380 nm) for DAPI and a B2 filter (blue light; λ = 465–496 nm) for Alexa Fluor^®^ 488. All images in each experimental series of a given experiment, using the specific antibodies, were captured at identical integration times with a DS-Fi1 CCD camera (Nikon, Tokyo, Japan). Under these conditions, negative control samples—incubated only with the secondary antibody conjugated to Alexa Fluor^®^ 488, without the primary antibody—did not produce a detectable signal, appearing completely dark in the images. During the analysis of nuclei exhibiting epigenetic modifications, the cell cycle phase of each nucleus (G1, S, or G2) was estimated by measuring nuclear DNA content using microfluorimetric analysis after DAPI staining. Quantitative analyses and fluorescence intensity (FI) measurements were performed in ImageJ software ver. 1.54p after conversion of the color images to grayscale and were expressed in arbitrary units [a.u.] as the mean pixel value (p.v.) covering the range from 0 (dark) to 255 (white) according to the methods described [154,155]. Standard background subtraction was applied by subtracting the average signal from areas lacking specific fluorescence.

### 4.7. Statistical Analysis

Data were analyzed using GraphPad Prism version 10 software (GraphPad Software, Boston, MA, USA). Before selecting the appropriate statistical test, data normality was assessed using the Shapiro–Wilk test. For datasets meeting the assumptions of normality, one-way analysis of variance (ANOVA) was performed, followed by Tukey’s post hoc test to evaluate differences between groups. For datasets not meeting normality assumptions, the non-parametric Kruskal–Wallis test with appropriate post hoc analysis was applied. In data presentation, variables with a normal distribution are reported as mean ± standard deviation (SD), whereas variables not meeting normality assumptions are presented as median with confidence interval (CI). In all analyses, the significance level was set at *p*  <  0.05. Statistical significance in figures is indicated with asterisks (**** *p* < 0.0001, *** *p* < 0.001, ** *p* < 0.01, * *p* < 0.05).

## 5. Conclusions

**Figure 10:**
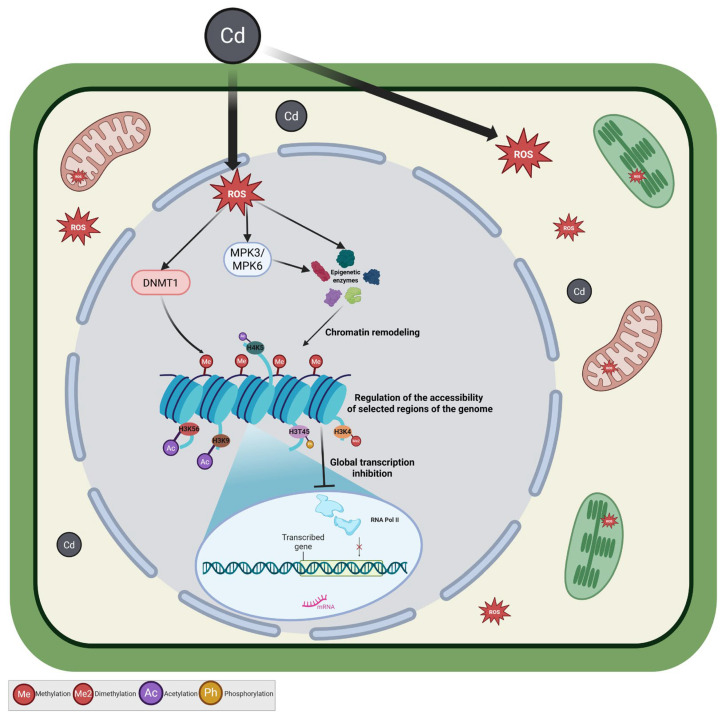
Proposed mechanism of cadmium (Cd) effects on the epigenetic regulation of gene expression. Arrows indicate the direction of processes or signaling pathways; arrows with pointed tips denote activation or enhancement, whereas arrows with blunt (flat) tips denote inhibition or suppression. Cd penetrates the cell, leading to the overproduction of reactive oxygen species (ROS). Elevated ROS levels activate signaling pathways involving MPK3/MPK6 kinases, which influence the activity of epigenetic enzymes. This triggers chromatin remodeling and changes in histone modifications, including methylation (H3K4), acetylation (H4K5, H3K9, H3K56), and phosphorylation (H3T45). Consequently, chromatin accessibility is reduced, and global gene transcription is inhibited. Created with BioRender.com.

Our study shows that DNA methylation and histone modifications form an integral part of the epigenetic response to cadmium stress. The observed changes contribute to chromatin reorganization, which may simultaneously reduce basal transcriptional activity and permit the activation of stress-responsive genes (Figure 10). In this manner, epigenetic remodeling likely acts as a functional link between early stress signaling and downstream transcriptional outcomes.

**Figure 11:**
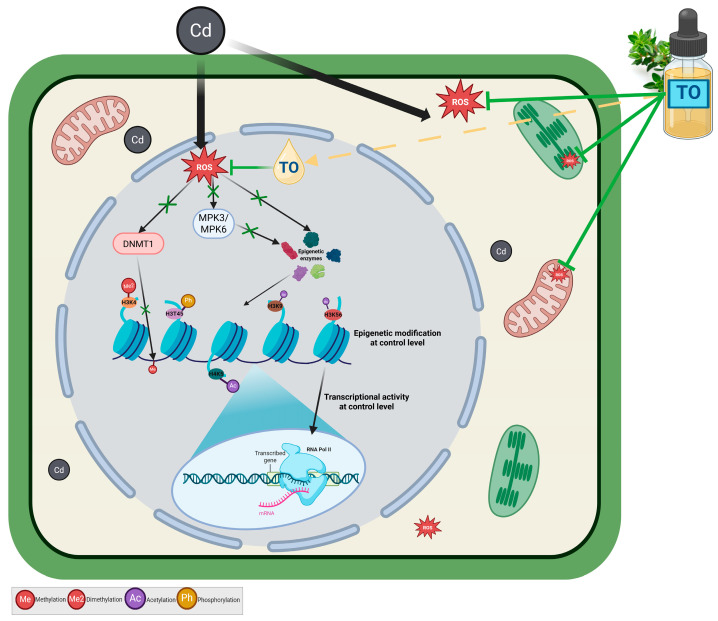
Proposed protective mechanism of thyme oil (TO) on cadmium (Cd)-induced epigenetic changes. Arrows indicate the direction of processes or signaling pathways; arrows with pointed tips denote activation or enhancement, whereas arrows with blunt (flat) tips denote inhibition or suppression. Exposure to Cd leads to the formation of reactive oxygen species (ROS), which disrupt the activity of epigenetic enzymes and MPK3/MPK6 kinases resulting in abnormal histone modifications (e.g., ↑H3K4Me2, ↓H3T45Ph, ↓H4K5Ac, ↑H3K9Ac, ↑H3K56Ac) and inhibition of global transcription. Treatment with TO reduces oxidative stress, restoring normal activity of epigenetic enzymes and maintaining balanced histone modifications. Consequently, chromatin structure is preserved and gene transcriptional activity remains at control levels. Created with BioRender.com.

The results also suggest potential mechanisms by which TO may mitigate cadmium-induced stress through modulation of epigenetic plasticity and transcriptional regulation. Our findings are consistent with the notion that TO acts during early stages of the cellular response, limiting the activation of detrimental signaling pathways and preventing alterations in chromatin structure (Figure 11).

Together, these observations expand current knowledge on epigenetic regulation under heavy metal stress and indicate that natural bioactive substances may contribute to strategies aimed at enhancing plant resilience in contaminated environments.

The presented data also support a broader perspective in which natural products such as TO, characterized by low toxicity, biodegradability, and high biological activity, may represent promising alternatives to synthetic agrochemicals. Essential oil-based formulations can be applied as foliar sprays, soil additives, or controlled-release systems such as microcapsules [156,157], in line with the principles of sustainable agriculture and the growing need to maintain crop performance under increasing heavy-metal pollution.

It should be emphasized that the present study was conducted exclusively on meristematic cells of *V. faba* exposed to a single cadmium concentration and a 24 h treatment with a defined TO dose. While this experimental design ensures clear and interpretable results, it also underscores the necessity for further investigation prior to practical application. Future studies should include a broader range of plant species, cadmium concentrations, and TO doses, as well as extended exposure periods, to better characterize dose- and time-dependent responses. From a molecular perspective, analysis of genes associated with oxidative stress responses and cell cycle regulation represents an important next step. Ultimately, comprehensive transcriptomic approaches combined with targeted validation of selected marker genes will enable more precise integration of physiological, biochemical, and epigenetic responses to cadmium toxicity and the protective action of TO.

## Data Availability

The raw data supporting the conclusions of this article will be made available by the authors on request.

## Abbreviations

The following abbreviations are used in this manuscript:

Cd	Cadmium
TO	Thyme oil
ROS	Reactive oxygen species
MAPK	Mitogen-activated protein kinases
ERK	Extracellular signal-regulated kinase
5-mC	5-methylcytosine
MET1	Methyltransferase 1
DNMT1	DNA (cytosine-5)-methyltransferase 1
CMT3	Chromomethylase 3
DRM2	Domains rearranged methylase 2
RdDM	RNA-directed DNA methylation
ROS1	Repressor of silencing 1
DME	Transcriptional activator Demeter
DML2	Demeter-like protein 2
DML3	Demeter-like protein 3
SUMO	Small ubiquitin-like modifier
HATs	Histone acetyltransferases
HDACs	Histone deacetylases
HMTs	Histone methyltransferases
